# Supplementary material for: Role of Maternal Diet in the Risk of Childhood Acute Leukemia: A Systematic Review and Meta-Analysis
Source: Int J Environ Res Public Health. 2023 Apr 6;20(7):5428. doi: 10.3390/ijerph20075428 (PMC10093835; doi:10.3390/ijerph20075428)
Supplement: Supplementary file 1 [file ijerph-20-05428-s001.zip › Supp Table S2. Quality assessment of the studies Fowkes.pdf]

Supplementary table S2. Quality assessment of the studies included into the systematic review evaluated by the checklist proposed by Fowkes et al.

Reference	Study design appropriate to objectives	Study sample representative					Control group acceptable				Quality of measurements and outcome				Completeness				Distorting influences				
	Objective: Cause	*Source of sample*	*Sampling method*	*Sample size*	*Non respondents*	*Entry criteria/exclusion*	*Definition of controls*	*Source of controls*	*Matching/randomization*	*Comparable characteristics*	*Validity*	*Reproducibility*	*Blindness*	*Quality control*	*Compliance*	*Dropouts*	*Deaths*	*Missing data*	*Extraneous treatments*	*Contamination*	*Changes over time*	*Confounding factors*	*Distortion reduced by analysis*
Abudaowd et al., 2021	0	0	0	0	+	0	0	0	0	0	0	NA	NA	NA	NA	NA	NA	0	NA	NA	NA	+	0
Ajrouche et al., 2014	0	0	0	0	0	0	0	0	0	0	0	NA	NA	NA	NA	NA	NA	0	NA	NA	NA	+	0
Amigou et al., 2012	0	0	0	0	0	0	0	0	0	0	0	NA	NA	NA	NA	NA	NA	0	NA	NA	NA	+	0
Bailey et al., 2012	0	0	0	0	0	0	0	0	0	0	0	NA	NA	NA	NA	NA	NA	0	NA	NA	NA	+	0
Bonaventure et al., 2013	0	0	0	0	0	0	0	0	0	0	0	NA	NA	NA	NA	NA	NA	0	NA	NA	NA	+	0
Bonaventure et al., 2015	0	0	0	0	0	0	0	0	0	0	0	NA	NA	NA	NA	NA	NA	0	NA	NA	NA	+	0
Clavel et al., 2005	0	0	0	0	0	0	0	0	0	0	0	NA	NA	NA	NA	NA	NA	0	NA	NA	NA	+	0
Dockerty et al., 2007	0	0	0	0	+	0	0	0	0	0	0	NA	NA	NA	NA	NA	NA	0	NA	NA	NA	+	0
Jensen et al., 2004	0	0	0	0	0	0	0	0	0	+	0	NA	NA	NA	NA	NA	NA	0	NA	NA	NA	+	0
Karalexy et al., 2019	0	0	0	0	0	0	0	0	0	0	0	NA	NA	NA	NA	NA	NA	0	NA	NA	NA	0	0
Kwan et al., 2006	0	0	0	0	0	0	0	0	0	0	0	NA	NA	NA	NA	NA	NA	0	NA	NA	NA	+	0
Kwan et al., 2009	0	0	0	0	0	0	0	0	0	0	0	NA	NA	NA	NA	NA	NA	0	NA	NA	NA	+	0
Linabery et al., 2010	0	0	0	0	+	0	0	0	0	0	+	NA	NA	NA	NA	NA	NA	0	NA	NA	NA	+	0
Madsen et al., 2020	0	0	NA	NA	NA	0	NA	NA	NA	NA	0	NA	NA	NA	NA	NA	NA	0	NA	NA	NA	0	0
McKinney et al., 1999	0	0	0	0	0	0	0	0	0	0	0	NA	NA	NA	NA	NA	NA	0	NA	NA	NA	+	0
Menegaux et al., 2005	0	0	0	0	0	0	0	0	0	0	0	NA	NA	NA	NA	NA	NA	0	NA	NA	NA	+	0
Menegaux et al., 2007	0	0	0	0	0	0	0	0	0	0	+	NA	NA	NA	NA	NA	NA	0	NA	NA	NA	+	0
Metayer et al., 2014	0	0	0	0	0	0	0	0	0	0	0	NA	NA	NA	NA	NA	NA	0	NA	NA	NA	0	0
Milne et al., 2010	0	0	0	0	0	0	0	0	0	0	0	NA	NA	NA	NA	NA	NA	0	NA	NA	NA	+	0
Milne et al., 2011	0	0	0	0	0	0	0	0	0	0	0	NA	NA	NA	NA	NA	NA	0	NA	NA	NA	+	0

Reference	Study design appropriate to objectives	Study sample representative					Control group acceptable				Quality of measurements and outcome				Completeness				Distorting influences				
	Objective: Cause	*Source of sample*	*Sampling method*	*Sample size*	*Non respondents*	*Entry criteria/exclusion*	*Definition of controls*	*Source of controls*	*Matching/randomization*	*Comparable characteristics*	*Validity*	*Reproducibility*	*Blindness*	*Quality control*	*Compliance*	*Dropouts*	*Deaths*	*Missing data*	*Extraneous treatments*	*Contamination*	*Changes over time*	*Confounding factors*	*Distortion reduced by analysis*
Milne et al., 2018	0	0	0	0	0	0	0	0	0	0	0	NA	NA	NA	NA	NA	NA	0	NA	NA	NA	0	0
Ognjanovic et al., 2011	0	0	0	0	+	0	0	0	0	0	+	NA	NA	NA	NA	NA	NA	0	NA	NA	NA	+	0
Orsi et al., 2015	0	0	0	0	0	0	0	0	0	0	0	NA	NA	NA	NA	NA	NA	0	NA	NA	NA	+	0
Peters et al., 1994	0	0	0	0	0	0	0	0	0	0	0	NA	NA	NA	NA	NA	NA	0	NA	NA	NA	+	0
Petridou et al., 1997	0	0	0	0	0	0	0	+	0	0	0	NA	NA	NA	NA	NA	NA	0	NA	NA	NA	+	0
Petridou et al., 2005	0	0	0	0	0	0	0	+	0	0	0	NA	NA	NA	NA	NA	NA	0	NA	NA	NA	+	0
Robison et al., 1989	0	0	0	0	0	0	0	0	0	0	0	NA	NA	NA	NA	NA	NA	0	NA	NA	NA	+	0
Ross et al., 1996	0	0	0	0	0	0	0	0	0	0	0	NA	NA	NA	NA	NA	NA	0	NA	NA	NA	+	0
Sarasua et al., 1993	0	0	0	++	0	0	0	0	0	0	0	NA	NA	NA	NA	NA	NA	0	NA	NA	NA	+	0
Schuz et al., 2007	0	0	0	0	0	0	0	0	0	0	+	NA	NA	NA	NA	NA	NA	0	NA	NA	NA	+	0
Shaw et al., 2004	0	0	0	0	0	0	0	0	0	0	+	NA	NA	NA	NA	NA	NA	0	NA	NA	NA	+	0
Singer et al., 2016	0	0	0	0	0	0	0	0	0	0	0	NA	NA	NA	NA	NA	NA	0	NA	NA	NA	+	0
Singer et al., 2016	0	0	0	0	0	0	0	0	0	0	0	NA	NA	NA	NA	NA	NA	0	NA	NA	NA	+	0
Thompson et al., 2001	0	0	0	0	0	0	0	0	0	0	0	NA	NA	NA	NA	NA	NA	0	NA	NA	NA	+	0
Van Steensel-Moll et al., 1985	0	0	0	0	0	0	0	0	0	0	0	NA	NA	NA	NA	NA	NA	0	NA	NA	NA	+	0
Wen et al., 2002	0	0	0	0	0	0	0	0	0	+	+	NA	NA	NA	NA	NA	NA	0	NA	NA	NA	+	0

Legend: 0=no flaw, +=minor flaw, ++=major flaw, NA= Not applicable.
